# Early Serum Metabolism Profile of Post-operative Delirium in Elderly Patients Following Cardiac Surgery With Cardiopulmonary Bypass

**DOI:** 10.3389/fnagi.2022.857902

**Published:** 2022-06-10

**Authors:** He Huang, Jingjing Han, Yan Li, Yonglin Yang, Jian Shen, Qiang Fu, Yu Chen

**Affiliations:** ^1^Department of Anesthesiology, First Affiliated Hospital of Nanjing Medical University, Nanjing, China; ^2^ Division of Infectious Diseases, Taizhou Clinical Medical School of Nanjing Medical University (Taizhou People’s Hospital), Taizhou, China; ^3^Nanjing Red Cross Blood Center, Nanjing, China

**Keywords:** metabolomics, cardiopulmonary bypass, perioperative neurocognitive disorder, post-operative delirium, lipid, phosphatidylinositol

## Abstract

**Background:**

Cardiac surgery with cardiopulmonary bypass (CPB) is considered to be one of the surgical types with the highest incidence of post-operative delirium (POD). POD has been associated with a prolonged intensive care and hospital stay, long-term neurocognitive deterioration, and increased mortality. However, the specific pathogenesis of POD is still unclear. Untargeted metabolomics techniques can be used to understand the changes of serum metabolites in early POD to discover the relationship between serum metabolites and disease.

**Materials and Methods:**

The present study recruited 58 elderly patients undergoing cardiac surgery with CPB. Serum was collected within the first 24 h after surgery. The Confusion Assessment Method (CAM) and ICU-CAM assessments were used to identify patients who experienced POD. All patients with normal post-operative cognitive assessment were included in the non-POD groups. Moreover, we collected serum from 20 healthy adult volunteers. We performed untargeted analyses of post-operative serum metabolites in all surgical groups, as well as serum metabolites in healthy non-surgical adults by using liquid chromatography mass spectrometry (LC/MS) and analyzed metabolic profiles and related metabolites.

**Results:**

The probability of POD after cardiac surgery were 31%. There were statistically significant differences in post-operative mechanical ventilation time, ICU stay time and post-operative hospital stay between POD and non-POD group (*P* < 0.05). And ICU stay time was an independent risk factor for POD. The analysis revealed that a total of 51 differentially expressed metabolites (DEMs) were identified by comparing the POD and non-POD group, mostly lipids and lipid-like molecules. Three phosphatidylinositol (PI) were down-regulated in POD group, i.e., PI [18:0/18:2 (9Z, 12Z)], PI [20:4 (8Z, 11Z, 14Z, 17Z)/18:0], and PI [18:1 (9Z)/20:3 (8Z, 11Z, 14Z)]. The receiver operating characteristic (ROC) curve analysis showed that three kinds of PI metabolites had the highest area under the curve (AUC), which were 0.789, 0.781, and 0.715, respectively. Correlation analysis showed that the expression of three PIs was negatively correlated with the incidence of POD.

**Conclusion:**

Our findings suggest that lipid metabolism plays an important role in the serum metabolic profile of elderly patients with POD in the early post-operative period. Low serum lipid metabolic PI was associated with incidence of POD in elderly following cardiac bypass surgery, which may provide new insights into the pathogenesis of POD.

## Introduction

Cardiopulmonary bypass (CPB) cardiac surgery is considered one of the surgical types with the highest incidence of post-operative delirium (POD) (Mailhot et al., 2019). Previous studies have shown that POD occurs in up to 50% of patients after cardiac surgery, and the main clinical symptoms include anxiety, mental disorder, decreased attention and cognitive decline (Pedemonte et al., 2020). Current findings suggest that perioperative enhanced neuroinflammatory responses, alterations in neurotransmitter and neuronal network function, and abnormal deposition of β-amyloid protein (Aβ) are closely related to the development of POD (Xie et al., 2014; Neerland et al., 2016).

The pathogenesis of POD in cardiac surgery patients still remains an unresolved problem. Numerous pre-operative comorbidities, hypoxic-ischemic damage in the brain induced by the CPB process, and long-term mechanical ventilation are independent risk factors for POD after cardiac surgery (Tse et al., 2015). POD has been associated with a prolonged intensive care and hospital stay, long-term neurocognitive deterioration, and increased mortality (Bhamidipati et al., 2017). Identifying the predictors of POD is important for the prevention and early treatment of this condition (Hayhurst et al., 2016). Therefore, exploring the pathogenesis and early prevention strategies of POD after cardiac surgery with CPB have become a serious medical and social problem.

Metabolomics is a detection technique for identifying and quantifying small molecule metabolites, which is widely regarded as the omics discipline closest to phenotype. It analyzes small molecule metabolites in body fluids, tissues and cells to obtain differential metabolites and then search for their related metabolic pathways. It is often used as one of the important means to identify biomarkers and pathways (Fiehn, 2016). Metabolomics has the advantages of high throughput, high accuracy, high sensitivity and non-invasion and can quantitatively reflect the dynamic response of biological systems to pathological stimulation, genetic modification and environmental impact (Johnson et al., 2016). The changes of metabolites in different samples can be detected by metabolomics, including cerebrospinal fluid, tissue, urine and serum, which has provided a novel perspective to investigate the mechanisms of neurodegenerative diseases, such as Alzheimer’s disease (AD) (Toledo et al., 2017). In recent years, metabolomics has been gradually applied to explore the pathogenesis and potential biomarkers of POD patients in non-cardiac surgery (Guo et al., 2019). Spermidine and putrescine and their metabolite precursor glutamine can be regarded as predictive markers of POD. However, to date, there has been no study on the use of metabolomics to observe the serum metabolic profile for POD after cardiac surgery.

In this study, we first analyzed the clinical characteristics of elderly patients with POD after cardiac surgery with CPB, and analyzed the risk factors by logistic regression model. Then, we analyzed the serum metabolic profiles of these patients using untargeted metabolomics of liquid chromatography mass spectrometry (LC/MS) and find closely related metabolic pathways and metabolites. These results may help us to find new pathogenesis of POD from the perspective of metabolism, and then facilitate the development of strategies for early intervention of these metabolic disturbances to prevent POD.

## Materials and Methods

### Patients and Setting

The protocols of this study were reviewed and approved by the Ethics Committee of the Medical Institution of the First Affiliated Hospital of Nanjing Medical University (2020-SR-329) and registered at the Chinese Clinical Trial Registry (ChiCTR2000037636). Written informed consent was obtained from all participants. The purpose of present study was to observe the plasma metabolic spectrum of the old patients undergoing CPB cardiac surgery, it is hard to define the normal range of different metabolites. Therefore, it is difficult to accurately estimate the sample size in this study. Referring to previous literature reports on the use of metabolomics to predict or diagnose related diseases (Peña-Bautista et al., 2019) and combined with related studies on perioperative neurological complications of cardiac surgery (Holmgaard et al., 2019), a preliminary sample size of 100 cases was estimated. Considering the possibility of drop out rate was 10% in the clinical trial, we therefore plan to enroll no fewer than 110 patients in this study.

**FIGURE 1:**
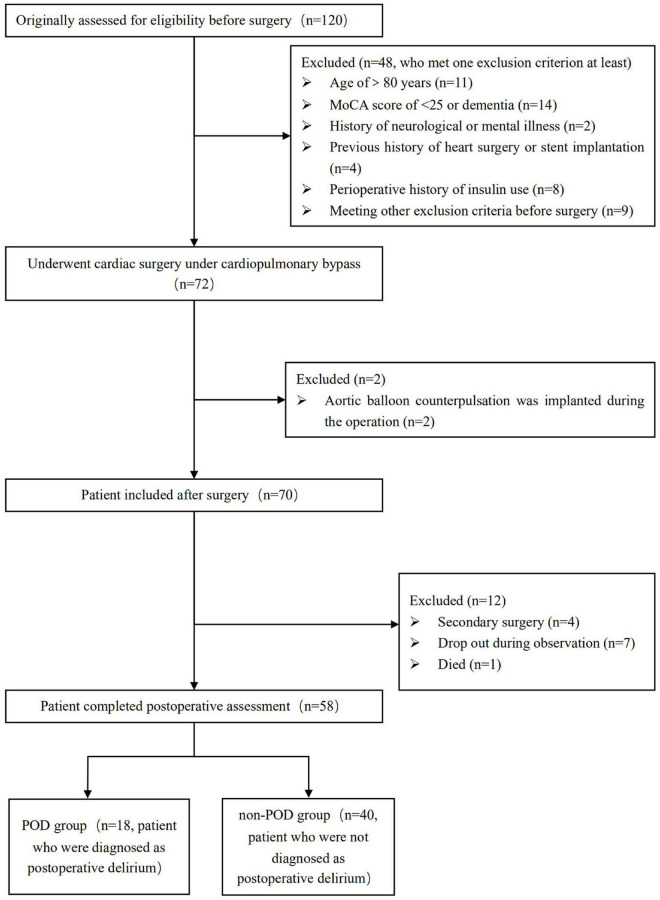
Flowchart showed the patient selection process in our matched case-control study.

As shown in Figure 1, a total of 58 patients who undergoing cardiac surgery with CPB in the First Affiliated Hospital of Nanjing Medical University from January 2020 to December 2020 were enrolled in this study. The inclusion criteria included patients between 65 and 80 years old, American Society of Anesthesiologists (ASA) physical status of II-III and New York Heart Association grade of II-III. All patients had a normal ability to hear, read and cooperate with neuropsychological tests. Exclusion criteria: the pre-operative Montreal Cognitive Assessment (MoCA) score was less than 25, patients with dementia, history of neurological or psychiatric disease, hospital with anxiety depression scale (HADS) over eight points, currently use sedatives or antidepressants, patients with liver and kidney dysfunction, patients with a history of cardiac surgery. In addition, 20 healthy adult volunteers with normal cognitive function, aged 60–70 years, were recruited as controls. The primary endpoint of this study was the identification of POD after cardiac surgery and associated analysis of early plasma metabolites. The secondary endpoints included analysis of risk factors for POD and comparison of post-operative complications.

### Anesthesia Management

During the operation, heart rate (HR), electrocardiogram (ECG), pulse oxygen saturation (SpO_2_), and invasive arterial blood pressure (IABP) were regularly monitored. Anesthesia was induced by midazolam (0.05–0.1 mg/kg), fentanyl (10–15 ug/kg), etomidate (0.3 mg/kg) and cisatracurium (0.15 mg/kg), and maintained with sevoflurane and propofol. The end-tidal CO_2_ (ETCO_2_) was maintained at 35–45 mmHg. A sedline sedation monitoring system (Masimo) was used to adjust the depth of anesthesia, and the patient state index (PSI) was maintained at 25–50% (Kim et al., 2018). Non-invasive regional cerebral oxygen saturation (rScO_2_) monitoring was performed, and the intraoperative rScO_2_ values of patients were maintained above 55% and not lower than 80% of baseline.

### Cardiopulmonary Bypass Management

Cardiopulmonary bypass (CPB), cardioplegia, and surgical procedures were performed according to standard guidelines. The perfusion flow during CPB was 2.2–2.6 L⋅min^–1^⋅m^–2^, the body temperature was controlled at 30–32°C, and hematocrit was maintained at above 20%. Hemodynamics were maintained with vasoactive drugs and antiarrhythmic drugs, and the mean arterial pressure was maintained at 60–80 mmHg. At the end of CPB, heparin was neutralized with protamine after CPB.

### Neurocognitive Function Assessment

Post-operative delirium assessment was performed by two attending physicians after professional training. We began the delirium assessment at 24 h after surgery, and the assessment was performed twice daily (08:00 and 20:00, ended on the 7th post-operative day). The Confusion Assessment Method (CAM) and ICU-CAM assessments were used to identify patients who experienced delirium based on four diagnostic criteria (Sauër et al., 2017): (1) acute morbidity and fluctuating changes of illness; (2) inattention; (3) thinking disorder; and (4) changes in consciousness level. After the presence or absence of each CAM feature was determined, CAM diagnoses were used to determine the presence of delirium. First, sedation was assessed using the Richmond Agitation-Sedation Scale (RASS), and for patients who had not removed endotracheal catheters or were unable to respond, the CAM-ICU was used for assessment. For those patients who could respond, CAM was used for evaluation (Chi et al., 2017).

### Blood Sample Collection

Venous blood (2 ml) was collected from all patients within 24 h after surgery. The blood sample was placed in a 5 ml test tube at 4°C for 30 min, followed by centrifugation at 3,000 rpm for 15 min at 4°C. After centrifugation, the serum was labeled with patient information and immediately stored at −80°C (Haier, China). Serum from healthy adult volunteers was collected on an empty stomach in the morning, and blood samples were treated in the same way.

### Serum Sample Preparation, Liquid Chromatography-Mass Spectrometry Analysis and Identification of Metabolic Profiles

For LC/MS analysis, samples stored at 80°C were thawed at room temperature. One hundred microliters of sample was added to a 1.5 ml centrifuge tube with 10 μl of 2 chloro-L-phenylalanine (0.3 mg/mL) dissolved in methanol as an internal standard, and the tube was vortexed for 10 s. Subsequently, 300 μl of an ice-cold mixture of methanol and acetonitrile (2:1 v/v) was added, and the mixture was vortexed for 1 min, ultrasonicated at ambient temperature (25 to 28°C for 10 min), and stored at 20°C for 30°min. The extract was centrifuged at 13,000 rpm and 4°C for 15 min. A 300 μl mixture of methanol and water (1:4 vol/vol) was added to each sample, vortexed for 30 s, and then placed at 4°C for 2 min. Samples were centrifuged at 13,000 rpm and 4°C for 5 min. The supernatants (150 μl) from each tube were collected using crystal syringes, filtered through 0.22 μm microfilters and transferred to LC vials. The vials were stored at −80°C until LC-MS analysis.

The ACQUITY UPLC I-Class system (Waters Corporation, Milford, CT, United States) was equipped with VION IMS QTOF Mass spectrometer (Waters Corporation, Milford, CT, United States) which used to analyze the metabolic profiling in both ESI positive and ESI negative ion modes. An ACQUITY UPLC BEH C 18 column (1.7 μm, 2.1 mm × 100 mm) was used for separation of all samples, and the column temperature was set at 45°C. The flow rate was 0.4 ml/min, and the mobile phases were 0.1% acetic acid in water (A) and acetonitrile (B). The compound separation was carried out under the following gradient program (time, % B): 0 min, 1% B; 1 min, 30% B; 2.5 min, 60% B; 6.5 min, 90% B; 8.5 min, 100% B; 10.7 min, 100% B; 10.8 min, 1% B and 13 min, 1% B. The sample injection volume was 1 μl. The mass spectrum parameters of the two modes are the same, as follows: electrospray capillary voltage: 2500V; injection voltage:4eV; ion source temperature:115°C; desolvation gas temperature: 450°C; desolvation gas flow:900 L/h; m/z ranges: 50–1000; scantime:0.2 s; interscan delay:0.02 s.

### Statistical Analyses

Data were statistically analyzed using SPSS 26.0 (SPSS, Chicago, IL, United States). Data are presented as the mean ± SD/median (interquartile spacing) or frequencies with percentages. If the assumptions of normality were met, then comparisons between the different study groups were performed using the independent samples’ *t*-test; otherwise, The Mann–Whitney *U*-test was used. The chi-square test or Fisher’s exact test was utilized to compare qualitative data, and *P* < 0.05 was considered to be statistically significant. Application of Logistic back Regression model was used to analyze related risk factors and calculate the OR value and 95% CI (*P* < 0.05).

Orthogonal partial least squares discriminant analysis (OPLS-DA) was implemented to visualize the differences in metabolites. The differential metabolites were selected on the basis of the combination of a statistically significant threshold of variable influence on projection values obtained from the OPLS-DA model and *p*-values from a two tailed Student’s *t*-test on the normalized peak areas, where metabolites with Variable important in projection (VIP) values larger than 1.0 and *p*-values less than 0.05 were considered differential metabolites. Metabolites were identified by Progenesis QI (Waters Corporation, Milford, CT, United States) Data Processing Software based on public databases, and the Kyoto Encyclopedia of Genes and Genomes (KEGG) database was used to analyze the metabolic pathway enrichment of differential metabolites. The sensitivity and specificity of target metabolite screening were analyzed by Further receiver operating characteristic (ROC) curve analysis based on a logistic regression model.

## Results

### Patient Characteristics

In the present study, a total of 120 participants were enrolled. After perioperative exclusion, a total of 58 patients undergoing cardiac surgery with CPB were eventually enrolled in this study (Figure 1). The findings revealed that 18 patients were diagnosed with POD (POD group), and 40 patients were not diagnosed with POD (non-POD group). POD after CPB surgery occurred in 31% of patients. As shown in Table 1, there were no significant differences in general characteristics among the POD and non-POD groups, such as education level, NYHA classification and MOCA score before the operation (*P* > 0.05).

**TABLE 1:** Demographic characteristics of the non-POD and POD group.

Characteristics	Non-POD group (*n* = 40)	POD group (*n* = 18)	*P*-value
Age (y)	69.25 ± 2.92	68.72 ± 2.93	0.867
Male	28 (70%)	15 (83.3%)	0.283
BMI	23.04 ± 3.06	24.61 ± 3.02	0.600
NYHA			0.493
II	14 (35%)	8 (44.4%)	
III	26 (65%)	10 (55.6%)	
EF	63.05 (8.6)	61.45 (10.65)	0.166
Hypertension	24 (60%)	10 (55.6%)	0.751
Coronary heart disease	6 (15%)	5 (27.8%)	0.251
Diabetes	8 (20%)	8 (20%)	0.054
Smoking within 1 year	4 (10%)	3 (16.7%)	0.567
Pre-operative hemoglobin (g/L)	131.5 (25.25)	136 (22.25)	0.274
Pre-operative albumin (g/L)	38.15 (5.15)	38.85 (7.63)	0.444
Education (<6 years)	12 (30%)	6 (33.3%)	0.800
Pre-operative MoCA	27.5 (2.0)	27.6 (3.25)	0.330

*POD, post-operative delirium; BMI, body mass index; NYHA, New York heart association; MoCA, Montreal cognitive assessment; EF, ejection fraction.*

### Comparison of Perioperative Information

The perioperative clinical findings demonstrated that there was no significant difference between the non-POD and POD groups, such as the type of cardiac surgery, anesthesia time, surgery time, CPB time, and the amount of intraoperative fluid infusion (Table 2). The post-operative mechanical ventilation time, ICU care duration and Length of hospital stay between the POD and non-POD groups showed statistically significant differences. The POD group had a significantly longer post-operative mechanical ventilation time, ICU stay time and post-operative hospital stay than non-POD group (Table 2). The post-operative follow-up results showed that there were no statistically significant differences in post-operative complications or scores of daily living ability 3 months after the operation between the two groups (Tables 2, 3). Considering the interaction and influence of each risk factor, all the risk factors with statistical significance in univariate analysis were included in multivariate Logistic regression analysis, our findings suggested that post-operative ICU stay time was an independent risk factor for POD (Table 4).

**TABLE 2:** Anesthesia and surgical data of the non-POD group and POD group.

Characteristics	non-POD group (*n* = 40)	POD group (*n* = 18)	*P*-value
Operation types			0.382
Valvular surgery	31 (77.5%)	14 (77.8%)	
OP-CABG	3 (7.5%)	3 (16.7%)	
Repair of ventricular septal defect	6 (15%)	1 (5,6%)	
Anesthesia duration (h)	6.23 (1.41)	5.61 (2.19)	0.391
Surgical duration (h)	5.33 (1.50)	5.25 (1.93)	0.460
CPB duration (h)	2.46 (1.13)	2.28 (1.14)	0.573
Aortic occlusion time (h)	1.91 ± 0.71	1.74 ± 0.65	0.702
Post-operative hemoglobin (g/L)	104.50 (20.50)	104.00 (28.25)	0.699
Post-operative albumin (g/L)	38.15 (5.15)	38.85 (7.63)	0.330
mechanical ventilation time (d)	1 (0)	2 (3.25)	0.001*
ICU care duration (d)	3 (2.75)	5 (7.25)	0.006*
Length of hospital stay (d)	10 (6.75)	14.5 (15.5)	0.003*
Post-operative ADL score			0.395
>95 score	38 (95%)	16 (88.9%)	
≤95 score	2 (5%)	2 (11.1%)	

OP-CABG, on-pump coronary artery bypass grafting; CPB, cardiopulmonary bypass; ICU, intensive care unit; ADL, activity of daily living. *p < 0.05.

**TABLE 3:** Comparison of other post-operative complications among the non-POD and POD group.

Characteristics	Non-POD group (*n* = 40)	POD group (*n* = 18)	*P*-value
Atrial arrhythmia	5 (12.5%)	3 (16.7%)	0.670
Acute respiratory insufficiency	6 (15%)	3 (16.7%)	0.871
Acute cerebral infarction	1 (2.5%)	2 (11.1%)	0.171
Acute renal insufficiency	2 (5%)	1 (5.6%)	0.930

**TABLE 4:** Multivariate logistic analysis of risk factors associated with post-operative delirium.

Effect	B	SE	Wald	P	OR	95% CI
ICU care duration	0.232	0.091	6.551	0.010*	1.262	1.056–1.507
Age	0.031	0.032	0.927	0.336	1.031	0.969–1.098
Mechanical ventilation time	0.291	0.262	1.227	0.268	1.337	0.800–2.236
Length of hospital stay	0.038	0.043	0.791	0.374	1.039	0.955–1.131

*p < 0.05.

### Overall Metabolic Profile of the Post-operative Delirium and Non-post-operative Delirium Groups After Cardiac Surgery

**FIGURE 2:**
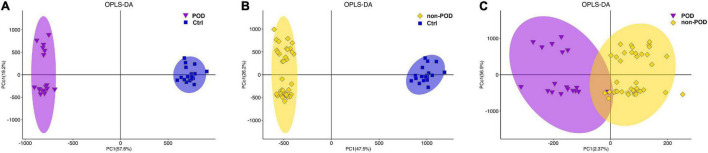
Orthogonal partial least squares discriminant analysis (OPLS-DA) score plot reveal the overall distribution of serum samples in POD and non-POD group. **(A)** OPLS-DA of metabolomics data from POD and healthy controls. **(B)** OPLS-DA of metabolomics data from non-POD and healthy controls. **(C)** OPLS-DA was used to distinguish differentially expressed metabolites (DEMs) between POD and non-POD group. The indicated groups are presented by different colors (blue: Ctrl; purple: POD; yellow: non-POD). POD: post-operative delirium. non-POD: non-post-operative delirium. Ctrl: healthy controls.

In multivariate statistical analysis, OPLS-DA method was used to observe the overall distribution of samples in POD and non-POD groups compared with the control group. The high degree of aggregation of the healthy control group demonstrated high stability of the LC/MS system during the whole sequence (Figures 2A,B). OPLS-DA scores indicated the trend of intragroup aggregation and intergroup separation. The scores of the OPLS-DA plot showed the differentially expressed metabolites (DEMs) between POD group and non-POD group (Figure 2C), suggesting there was significant intragroup aggregation and intergroup difference between the POD and non-POD groups.

### Differentially Expressed Metabolites and Metabolic Pathways in Post-operative Delirium Group Compared With Non-post-operative Delirium Group

**FIGURE 3:**
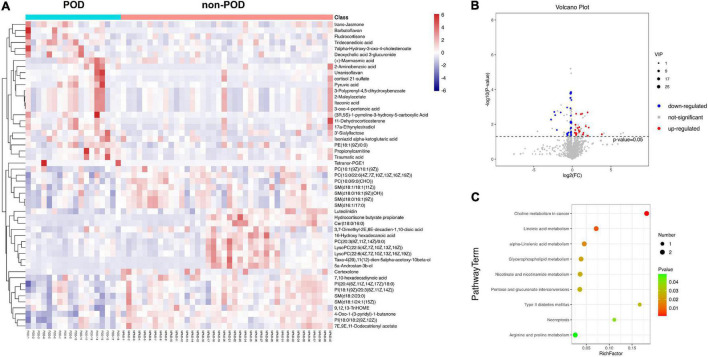
**(A)** A heatmap showed the 51 differentially expressed metabolites (DEMs, *P* < 0.05; variable influence on projection >1) between POD and non-POD patients after cardiac surgery. The color, ranging from blue to red, indicated that the expression abundance of metabolites ranged from low to high; that is, red indicated a higher expression abundance of different metabolites. **(B)** Volcano plot represented the 51 DEMs when compared between POD and non-POD groups. Compared with non-POD group, blue dots represent down-regulated metabolites and red dots represent up-regulated metabolites in POD group. **(C)** The 51 differentially expressed metabolites were subjected to Kyoto Encyclopedia of Genes and Genomes (KEGG) pathway enrichment analyses, and the bubble diagram showed a total of nine significantly different pathways (*P* < 0.05).

In our study, the statistical analysis revealed that a total of 51 DEMs were identified by comparing the POD group and non-POD group (VIP > 1 and *P* < 0.05). The names of the 51 screened metabolites are shown in Supplementary Table 1. Hierarchical clustering analysis was conducted for the expression levels of all DEMs, and DEMs was visualized through a heatmap (Figure 3A). The *P*-value, VIP and fold change values of the screened DEMs were visualized by a volcano map, 24 metabolites were up-regulated and 27 metabolites were down-regulated in the serum of POD patients (Figure 3B).

After further analysis of 51 DEMs, 38 metabolites were lipids and lipid-like molecules. The 51 DEMs were subjected to KEGG pathway enrichment analyses, and the bubble diagram of the enrichment pathway was shown in Figure 3C. A total of 9 pathways showed statistically significant differences (*P* < 0.05). The top four metabolic pathways were lipid-related metabolic pathways, i.e., choline metabolism, linoleic acid metabolism alpha-Linolenic acid metabolism and glycerophospholipid metabolism. The remaining five metabolic pathways were also closely related to lipid metabolism, namely nicotinate and nicotinamide metabolism, pentose and glucuronate interconversions, type II diabetes mellitus, necroptosis, arginine and proline metabolism. It was suggested that abnormal lipid metabolism played some role in the pathogenesis of POD after cardiac surgery.

### Phosphatidylinositol Associated With Increased Risk of Post-operative Delirium After Cardiac Surgery in Elderly Patients

**FIGURE 4:**
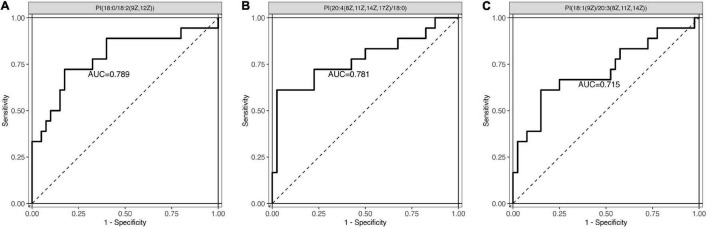
Receiver operating characteristic (ROC) curve analysis was used to evaluated the specificity and sensitivity of these three potential biomarkers. **(A)** PI [18:0/18:2 (9Z, 12Z)]: the area under the curve (AUC) was 0.789. **(B)** PI [20:4 (8Z, 11Z, 14Z, 17Z)/18:0]: the AUC was 0.781. **(C)** PI [18:1 (9Z)/20:3 (8Z, 11Z, 14Z)]: the AUC was 0.715. PI, phosphatidylinositol.

**FIGURE 5:**
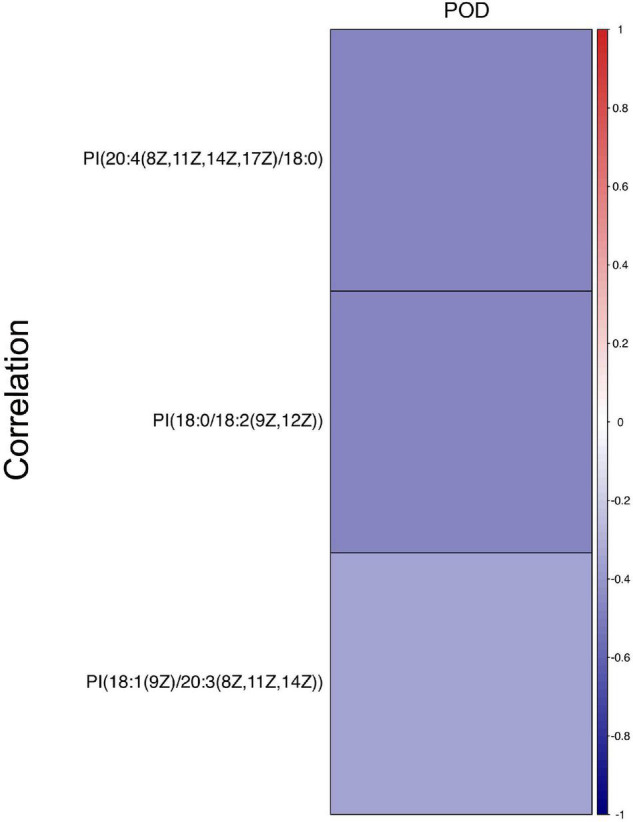
Correlation analysis between three PIs and the incidence of POD after cardiac surgery. Different colors represent different correlation coefficients, red is positive, indicating that the two are positively correlated. Blue is negative, indicating a negative correlation. The present findings suggested that three PIs were negatively correlated with the incidence of POD after cardiac surgery.

Next, a total of 51 DEMs were analyzed by ROC curve analysis based on a logistic regression model to evaluated the specificity and sensitivity of these potential biomarkers. Compared with non-POD group, the expression of PI [18:0/18:2 (9Z, 12Z)], PI [20:4 (8Z, 11Z, 14Z, 17Z)/18:0] and PI (18:1 (9Z)/20:3 (8Z, 11Z, 14Z)] in POD group were significant decreased. The three kinds of PI metabolites had the highest areas under the curve (AUC), which were 0.789, 0.781, and 0.715, respectively (Figure 4). Correlation analysis also showed that the three kinds of PI metabolites were all negatively correlated with the incidence of POD, correlation coefficients were −0.479, −0.475, and −0.348, respectively (Figure 5). It was suggested that the three kinds of PI are closely related to POD development after cardiac surgery.

## Discussion

Despite prevention strategies based on best practice guidelines, up to 30–60% of patients undergoing cardiac surgery experience POD, a complication that can prolong ICU stays, complicate recovery and further increase the risk of death (Daiello et al., 2019). POD is a typical multifactorial disease, and its occurrence is related to the superposition and interaction between the susceptible factors of patients before admission and the factors during hospitalization (Cortés-Beringola et al., 2021). In this study, after we strictly controlled the patient enrollment, there were statistically significant differences in post-operative mechanical ventilation time, ICU stay time and hospital stay between the POD group and the non-POD group. After multivariate Logistic regression analysis, we found that post-operative ICU stay was an independent risk factor for POD. This may be related to the advanced age of the patient, the decline of post-operative functional status and other factors (Greaves et al., 2020). Therefore, effective perioperative evaluation and optimization, particularly of older people undergoing surgery, has proved to be very important (Jin et al., 2020; Alam and Ma, 2022).

The pathophysiology of delirium is thought to involve disruption of neurotransmitter signals and neuroinflammation (Maldonado, 2013). Post-operative neuroinflammation can lead to synaptic damage, neuronal dysfunction and death, and impaired neurogenesis. A variety of pro-inflammatory cytokines, such as TNFα, maintain a chronic neuroinflammatory state characterized by POD (Alam et al., 2018). Although several different pathways have been proposed to explain this disruption, the potential mechanisms still remain unclear. Currently, a critical hypothesis for the development of POD after cardiac surgery is systemic inflammatory response syndrome (SIRS) related to CPB (Zhu et al., 2021). SIRS contributes to leakage of the blood-brain barrier and the development of brain edema and inflammation and may play a key role in the pathogenesis of POD (Zhang et al., 2017). The stress response to cardiac surgery has also been investigated as a possible factor in the development of POD (McDonald et al., 2014). In addition, cardiac surgery may, to a certain extent, cause the formation of microemboli in the brain, cerebral hypoperfusion, decreased cerebral oxygen saturation, release of inflammatory factors and fluctuations in body temperature, and other pathological and physiological changes (Karu et al., 2010). In previous studies, Qian found that phosphatidylserine might be a biological marker for the diagnosis of post-operative cognitive dysfunction after non-cardiac surgery based on the metabolomics method (Qian and Wang, 2020). Phosphatidylserine is closely associated with the activation of the nitric oxide signaling pathway, PI3K-Akt signaling pathway and mTOR signaling pathway, and might be an important factor in the pathogenesis of POD. However, the relationship between Phosphatidylserine or other lipids and delirium after CPB has not been confirmed.

The purpose of this study was to identify the POD related serum DEMs and metabolic pathways by LC-MS untargeted metabolomics technology. The statistical analysis revealed that a total of 51 DEMs and 9 metabolic pathways were identified, mainly manifested as lipids and lipid metabolism disorders (VIP > 1 and *p* < 0.05). Lipids play a major role in the structure, function and physiology of neurons, such as neural communication, neurogenesis, synaptic transmission, signal transduction, membrane partitioning, and gene expression regulation (Cermenati et al., 2015). Studies have revealed that brain lipid peroxidation is an early event of AD, and more lipid granules (or fat inclusion bodies) are shown in the glial cells of AD patients, suggesting that abnormal lipid metabolism is one of the key pathogenesis mechanisms of neurodegenerative diseases (Karch and Goate, 2015). Genome-wide association studies have found an association between AD and several genes involved in lipid homeostasis, such as apolipoprotein E and sortilin-related receptor 1 (Marin et al., 2017).

The perioperative lipid metabolism imbalance may be regarded as a risk factor for neurodysfunction. The administration of the lipid-lowering drugs simvastatin and fenofibrate before cardiac surgery has been shown to prevent cognitive dysfunction and alteration of neuronal integrity induced by CPB. Simvastatin and fenofibrate both protect endothelial function and reduce inflammatory processes (Ouk et al., 2016). Meanwhile, studies have shown that the new docosahexaenoic acid-derived lipid mediator neuroprotective hormone D1 can reduce the incidence of POD (Yang et al., 2020). In a recent study, researchers recruited older patients with hip fracture who undergoing hemiarthroplasty and analyzed blood samples from POD patients using a metabolomics platform (Guo et al., 2017). They found that the levels of ω3 and ω6 fatty acids were lower in the POD group than in the non-POD group both before and after surgery. In addition, several lipid-related pathways have been confirmed to be closely related to the occurrence of POD. The changes in pre-operative activity of cholinergic biomarkers were associated with the development of POD in elderly patients (Lin et al., 2020). Abnormalities in these lipid metabolism pathways may increase the vulnerability of the brain, leading to POD.

In the present study, we found an important lipid, phosphatidylinositol (PI), may be a potential research focus in the pathogenesis of POD after cardiac surgery. Compared with non-POD group, PI [18:0/18:2 (9Z, 12Z)], PI [20:4 (8Z, 11Z, 14Z, 17Z)/18:0] and PI [18:1 (9Z)/20:3 (8Z, 11Z, 14Z)] were down-regulated in POD group. Correlation analysis showed that the three kinds of PI were negatively correlated with POD. The analysis of ROC curve area suggested that PI may closely associated with the development of POD. The PI signal transduction pathway is involved in regulating various functions of cells, such as cell growth, cell apoptosis, membrane transport, cytoskeleton regulation, neurotransmitter signal transduction, and calcium channel regulation in nerve tissue (Dall’Armi et al., 2013). A growing interest raised around the role of signal transduction systems in a number of human diseases including neurodegenerative diseases, especially in the systems related to the PI pathway and AD (Heras-Sandoval et al., 2014). Recently, a study of lipid changes in human cerebrospinal fluid (CSF) showed that the sulfonamide/ PI ratio may serve as a useful biomarker for identifying patients with AD, with a sensitivity of 90% and specificity of 100% (Huynh et al., 2020).

The PI3K/Akt signaling pathway, a key molecular signal transduction pathway composed of PI, is involved in several neurodegenerative diseases (Yang et al., 2018). For example, the ratio of phospho-Akt/total-Akt decreases in dopaminergic neurons, supporting the notion that Akt-mediated signaling pathways are suppressed in pathogenesis of Parkinson’s disease (Malagelada et al., 2008). Studies have confirmed that triggering a variety of upstream cytokines and proteins can activate the PI3K/Akt signaling pathway, thereby improving the neurological function and reducing neuroinflammation after brain injury (Zhu et al., 2018; Chen et al., 2020). Brain injury after CPB involves multiple mechanisms, including perioperative cerebrovascular embolism, decreased cholinergic neuronal activity, and neuroinflammation. PI3K/Akt signaling pathway plays an important role. At present, there is no associated study between PI and delirium after CPB. Based on the present findings, it is suggested that abnormal PI metabolism may play an important role in the development of POD after cardiac surgery. Meanwhile, in future studies, we will pay more attention to the specific mechanism of PI influencing POD by comparing various subtypes of PI in multiple periods, so as to provide more in-depth understanding of POD occurrence after CPB.

## Limitation

This study identified the differential metabolic profiles in patients; however, there are some limitations that need to be improved. First, the sample size was not sufficient to provide strong support for clinical practice and multicenter, large-sample clinical studies need to be performed in the future. Second, we only analyzed early post-operative metabolic changes in elderly patients with POD. In the next step, we need to conduct further validation studies on samples at different times before and after surgery. Third, the potential mechanism of lipid metabolism disorder after CPB cardiac surgery has not been clarified which might be beneficial for better exploring cognitive disorders.

## Conclusion

In summary, we used untargeted metabolomics techniques to analyze the metabolic profile changes in the serum of patients undergoing cardiac surgery with CPB in the early post-operative period. The disturbance of lipid metabolism may be an important mechanism in the occurrence of POD. Through further screening and ROC curve analysis, low serum lipid metabolic PI was associated with incidence of POD in elderly following cardiac bypass surgery. PI and its related metabolic pathways can be used as an important breakthrough point to study the development of POD in elderly patients, so as to find its specific pathogenesis.

## Data Availability Statement

The original contributions presented in the study are included in the article/Supplementary Material, further inquiries can be directed to the corresponding author/s.

## Ethics Statement

The studies involving human participants were reviewed and approved by the Ethics Committee of the Medical Institution of the First Affiliated Hospital of Nanjing Medical University (2020-SR-329). The patients/participants provided their written informed consent to participate in this study.

## Author Contributions

HH and JH were responsible for the overall study design, and data analysis, designed this review, searched the literature, and wrote the initial manuscript. YL and JS made the table and figures. YC, YY, and QF supervised and provided critical comments on the manuscript. All authors reviewed and approved the manuscript.

## Conflict of Interest

The authors declare that the research was conducted in the absence of any commercial or financial relationships that could be construed as a potential conflict of interest.

## Publisher’s Note

All claims expressed in this article are solely those of the authors and do not necessarily represent those of their affiliated organizations, or those of the publisher, the editors and the reviewers. Any product that may be evaluated in this article, or claim that may be made by its manufacturer, is not guaranteed or endorsed by the publisher.
