# Supplementary material for: Early Serum Metabolism Profile of Post-operative Delirium in Elderly Patients Following Cardiac Surgery With Cardiopulmonary Bypass
Source: Front Aging Neurosci. 2022 Jun 10;14:857902. doi: 10.3389/fnagi.2022.857902 (PMC9226449; doi:10.3389/fnagi.2022.857902)
Supplement: Supplementary file 1 [file Table_1.docx]

Supplementary Table 1 Fifty-one screened differentially expressed metabolites

| **Metabolites** | **Sub Class** | **VIP** | **P-value** | **log2(FC)** | **Trend** |
| --- | --- | --- | --- | --- | --- |
| SM(d18:0/16:1(9Z)) | Phosphosphingolipids | 11.844 | 0.008 | -0.170 | ↓ |
| PI(20:4(8Z,11Z,14Z,17Z)/18:0) | Glycerophosphoinositols | 8.865 | 0.000 | -0.214 | ↓ |
| SM(d16:1/17:0) | Phosphosphingolipids | 7.212 | 0.007 | -0.178 | ↓ |
| LysoPC(22:6(4Z,7Z,10Z,13Z,16Z,19Z)) | Glycerophosphocholines | 5.290 | 0.034 | -0.651 | ↓ |
| Itaconic acid | Fatty acids and conjugates | 5.087 | 0.036 | 0.506 | ↑ |
| PI(18:0/18:2(9Z,12Z)) | Glycerophosphoinositols | 5.052 | 0.000 | -0.170 | ↓ |
| SM(d18:1/18:1(11Z)) | Phosphosphingolipids | 4.716 | 0.029 | -0.183 | ↓ |
| 3-oxo-4-pentenoic acid | Fatty Acids and Conjugates | 4.393 | 0.034 | 0.515 | ↑ |
| trans-Jasmone | Carbonyl compounds | 4.238 | 0.033 | 0.515 | ↑ |
| PC(20:3(8Z,11Z,14Z)/0:0) | Glycerophosphocholines | 4.238 | 0.043 | -0.606 | ↓ |
| PC(15:0/22:6(4Z,7Z,10Z,13Z,16Z,19Z)) | Glycerophosphocholines | 3.940 | 0.016 | -0.16 | ↓ |
| SM(d18:2/23:0) | Phosphosphingolipids | 3.329 | 0.000 | -0.240 | ↓ |
| PI(18:1(9Z)/20:3(8Z,11Z,14Z)) | Glycerophosphoinositols | 2.800 | 0.007 | -0.162 | ↓ |
| LysoPC(22:5(4Z,7Z,10Z,13Z,16Z)) | Glycerophosphocholines | 2.452 | 0.038 | -0.656 | ↓ |
| Unanisoflavan | Flavonoids | 2.402 | 0.046 | 2.007 | ↑ |
| 11-Dehydrocorticosterone | Hydroxysteroids | 2.164 | 0.038 | 1.387 | ↑ |
| Tetranor-PGE1 | Unclassified | 2.120 | 0.037 | 3.831 | ↑ |
| Propionylcarnitine | Fatty acid esters | 2.102 | 0.015 | 0.906 | ↑ |
| PE(18:1(9Z)/0:0) | Glycerophosphoethanolamines | 2.077 | 0.011 | 0.421 | ↑ |
| Taxa-4(20),11(12)-dien-5alpha-acetoxy-10beta-ol | Isoprenoids | 2.035 | 0.005 | -2.742 | ↓ |
| 3-Polyprenyl-4,5-dihydroxybenzoate | Benzoic acids and derivatives | 2.009 | 0.041 | 0.494 | ↑ |
| 7alpha-Hydroxy-3-oxo-4-cholestenoate | Bile acids, alcohols and derivatives | 1.999 | 0.030 | 0.330 | ↑ |
| Deoxycholic acid 3-glucuronide | Steroidal glycosides | 1.998 | 0.016 | 0.690 | ↑ |
| 3'-Sialyllactose | Carbohydrates and carbohydrate conjugates | 1.994 | 0.021 | 0.380 | ↑ |
| Hydrocortisone butyrate propionate | Steroids | 1.965 | 0.002 | -2.325 | ↓ |
| SM(d18:1/24:1(15Z)) | Phosphosphingolipids | 1.723 | 0.042 | -0.159 | ↓ |
| 16-Hydroxy hexadecanoic acid | Fatty acids and conjugates | 1.721 | 0.030 | -0.485 | ↓ |
| Pyruvic acid | Alpha-keto acids and derivatives | 1.664 | 0.002 | 1.977 | ↑ |
| 9,12,13-TriHOME | Fatty acids and conjugates | 1.626 | 0.003 | -0.193 | ↓ |
| (+)-Marmasmic acid | Isoprenoids | 1.610 | 0.003 | 1.102 | ↑ |
| Traumatic acid | Fatty acids and conjugates | 1.579 | 0.022 | 0.827 | ↑ |
| 7E,9E,11-Dodecatrienyl acetate | Fatty esters | 1.537 | 0.000 | -0.119 | ↓ |
| 4-Oxo-1-(3-pyridyl)-1-butanone | Carbonyl compounds | 1.519 | 0.000 | -0.486 | ↓ |
| 7,10-hexadecadiynoic acid | Fatty Acids and Conjugates | 1.512 | 0.001 | -0.225 | ↓ |
| Cortexolone | Hydroxysteroids | 1.456 | 0.021 | -1.995 | ↓ |
| Isoniazid alpha-ketoglutaric acid | Pyridinecarboxylic acids and derivatives | 1.436 | 0.025 | 0.959 | ↑ |
| 17a-Ethynylestradiol | Estrane steroids | 1.424 | 0.002 | 1.274 | ↑ |
| Tridecanedioic acid | Fatty acids and conjugates | 1.409 | 0.002 | 0.497 | ↑ |
| 5a-Androstan-3b-ol | Androstane steroids | 1.390 | 0.003 | -2.214 | ↓ |
| PC(16:0/9:0(CHO)) | Unclassified | 1.307 | 0.026 | -0.150 | ↓ |
| PC(16:1(9Z)/16:1(9Z)) | Glycerophosphocholines | 1.300 | 0.036 | -0.176 | ↓ |
| Barbatoflavan | Flavonoids | 1.238 | 0.015 | 2.122 | ↑ |
| 2-Aminobenzoic acid | Benzoic acids and derivatives | 1.225 | 0.031 | 1.449 | ↑ |
| cortisol 21-sulfate | Sulfated steroids | 1.217 | 0.013 | 1.743 | ↑ |
| 3,7-Dimethyl-2E,6E-decadien-1,10-dioic acid | Unclassified | 1.215 | 0.048 | -0.527 | ↓ |
| Cer(t18:0/16:0) | Ceramides | 1.194 | 0.002 | -1.504 | ↓ |
| 2-Maleylacetate | Medium-chain keto acids and derivatives | 1.189 | 0.035 | 0.509 | ↑ |
| SM(d18:0/16:1(9Z)(OH)) | Phosphosphingolipids | 1.123 | 0.018 | -0.213 | ↓ |
| Fludrocortisone | Hydroxysteroids | 1.113 | 0.049 | 0.454 |  |
| (3R,5S)-1-pyrroline-3-hydroxy-5-carboxylic Acid | Amino acids, peptides, and analogues | 1.097 | 0.028 | 0.870 |  |
| Luteolinidin | Hydroxyflavonoids | 1.061 | 0.001 | -0.672 | ↓ |
